# WiPFIM: A digital platform for interlinking biocollections of wild plants, fruits, associated insects, and their molecular barcodes

**DOI:** 10.1002/ece3.11457

**Published:** 2024-06-01

**Authors:** Bonface Onyango, Robert Copeland, John Mbogholi, Mark Wamalwa, Caleb Kibet, Henri E. Z. Tonnang, Kennedy Senagi

**Affiliations:** ^1^ Data Management, Modeling and Geo‐Information (DMMG) Unit International Centre of Insect Physiology and Ecology Nairobi Kenya; ^2^ Biochemistry and Biotechnology Department Pwani University Kilifi Kenya

**Keywords:** biocollections, biodiversity, data integration, digitization, ecology, natural history collections, plants‐insect interaction

## Abstract

The current knowledge on insects feeding on fruits is limited, and some of the scarce existing data on the fruit‐associated insects are secluded within the host institutions. Consequently, their value is not fully realized. Moreover, in countries like Kenya, the integration of biocollections data within a digital framework has not been fully exploited. To address these gaps, this article presents a description of the development of a web‐based platform for data sharing and integrating biodiversity historical data of wild plants, fruits, associated insects, and their molecular barcodes (WiPFIM) while leveraging data science technologies. The barcodes corresponding to the biocollections data were retrieved from BOLD database. The platform is an online resource about fruit–insect interactions that can be of interest to a worldwide community of users and can be useful in building innovative tools. The platform is accessible online at https://test‐dmmg.icipe.org/wpfhi.

## Graphical abstract

This article presents a description of the development of a web‐based platform for data sharing and integrating biodiversity historical data of wild plants, fruits, associated insects, and their molecular barcodes (WiPFIM) while leveraging data science technologies. The platform holds invaluable potential in understanding fruit–insect interactions and can function as a decision‐making tool.

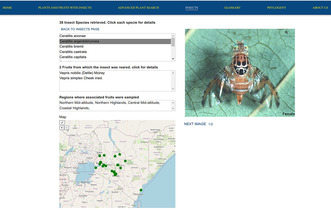

## 1 INTRODUCTION

Biocollections are important in ecological, taxonomic, and biogeographic studies. Biocollections are curated, preserved, and maintained by natural history museums or research institutions (Mulcahy et al., 2022). The museum biocollections and their associated metadata have been used to study species invasion and native species range shifts including dragonfly (Ball‐Damerow et al., 2015), spiders (Krehenwinkel & Tautz, 2013), butterflies (Kharouba et al., 2009), and grasshoppers (Berger et al., 2010). They are also an invaluable source of the historical occurrence of a given species in a particular geographical area (Weigelt et al., 2020).

Traditionally, researchers relied on morphological characteristics to identify and study biocollections. However, advances in molecular techniques have revolutionized the field. DNA barcoding and morphological traits have been used for species identification and biogeographic studies, each complementing the other. Although many biodiversity platforms focus solely on digitizing plant or insect biocollections, there is often a limited integration of molecular data. With recent advances in technologies and increased willingness to share data, most molecular barcodes are available in public databases like GenBank (NCBI, 2023), Barcode of Life (BOLD) (Ratnasingham & Hebert, 2007), and Coins database (Magoga, 2022). Morever, the advances in digital technology and bioinformatics tools have provided new opportunities for studying, analyzing, and sharing biocollections data (Heberling et al., 2019; Magoga et al., 2022; Meineke et al., 2018; Ponta et al., 2019; Wen et al., 2017).

Although bioinformatics tools such as MAFFT (Katoh & Standley, 2013) can construct phylogenetic trees using barcode sequences, these tools often produce phylogenetic trees in a file format that requires the use of distinct software such as phylotree.js (Shank et al., 2018) for visualization. The scattered nature of these tools limits their usage. Integrating these tools into a unified digital platform can allow users to explore both phylogenetic relationships and morphological information for biocollections. For example, softwares such as SHOOT.bio (Emms & Kelly, 2022) can allow users to search their query sequence against a database of gene families and provide a phylogenetic tree with the query sequence (Emms & Kelly, 2022).

The Global Biodiversity Information Facility (GBIF) (GBIF, 2022), BioBlitz (Society, 2022), The Biodiversity Collections Network (BCoN) (BCON, 2022), Herbaria@Home (Herbaria@Home, 2022), iNaturalist (iNaturalist, 2022), and Zooniverse (Zooniverse, 2022) are among the existing digital platforms for plant and insect biodiversity data. However, there are relatively inadequate photographs identifying both the plant and the associated insects (Gazdic & Groom, 2019). Previous studies have shown that despite the presence online of biodiversity databases like GBIF which house global data, their data is notably skewed towards regions on the northern latitudes and their records are still data‐deficient for some of the world's biodiversity hotspots, especially for Africa (Hochmair et al., 2020; Kharouba et al., 2018; Lendemer et al., 2019). A major issue with existing biodiversity platforms is data quality (Mugford et al., 2021) which may be compromised. For instance, in iNaturalist, the uploaded species are identified by platform participants who may have varying identifications of the uploaded images (Di Cecco et al., 2021).

To broaden the utilization of plants and insect biocollections, researchers at the International Centre of Insect Physiology (*icipe*) have undertaken investigations aimed at identifying wild fruit species serving as reservoir hosts for mainly pestiferous fruit flies (Tephritidae) in Kenya. The team collected fruit samples from various regions across Kenya and subjected them to controlled rearing to facilitate the emergence of insects. The data gathered for each fruit sample comprised geographical information, plant species details, and the insect species that emerged from the fruits of these plants. Despite the production of numerous published articles (Copeland, 2009; Copeland et al., 2002, 2009), the entirety of the dataset was not made accessible for scrutiny and analysis by researchers and the wider public.

Our study aimed to develop a digital platform for integrating morphological and geographic data of wild plants, fruits, and their associated insects hosted by *icipe* and linking them to their corresponding publicly available molecular barcodes. Through digitization, the scientific community can gain access to the resourceful biocollections data on interactions between fruits and insects without necessarily visiting Kenya or the host institution. In this platform, we have incorporated a phylogeny feature using publicly available molecular barcodes that were retrieved based on the plants and insects that were identified at the species level from the biocollection records.

## 2 MATERIALS AND METHODS

### 2.1 Dataset

This research builds on biocollections data from the study by Copeland et al. (2009). The biocollections are hosted by *icipe* and contain 873 records of wild plants, fruits, and their associated insect species in Kenya (Copeland et al., 2009). In the study by Copeland et al. (2009), the fruits were sampled either from plants or from the ground underneath them. Occasionally, binoculars were used to link fallen fruit with the ones still on trees, especially under tall trees. Leaves, stems, and flowers were pressed in the field and photographs were taken as documentation to collect only ripe fruit and avoid rotten ones. The fruit samples were stored in hanging polythene bags within plastic containers during transportation to avoid damage. In the laboratory, fruits were placed in rectangular plastic containers with holes, nested within larger containers filled with sand. A plastic cover with mesh replaced a section of the smaller container's lid. Fruits were stored for a maximum of 2 months, whereas adult insects were kept for 1–3 days before preservation. Due to the risk of contamination by common Drosophilidae species, these small flies were not linked to the fruit species they emerged from in the laboratory. Consequently, this fly family was not further examined. Since the biocollections were not sent for barcoding, we downloaded the barcode sequences from BOLD (Ratnasingham & Hebert, 2007) with IDs that matched both the plants and insects that were identified and had species name in the records.

### 2.2 System architecture

**FIGURE 1:**
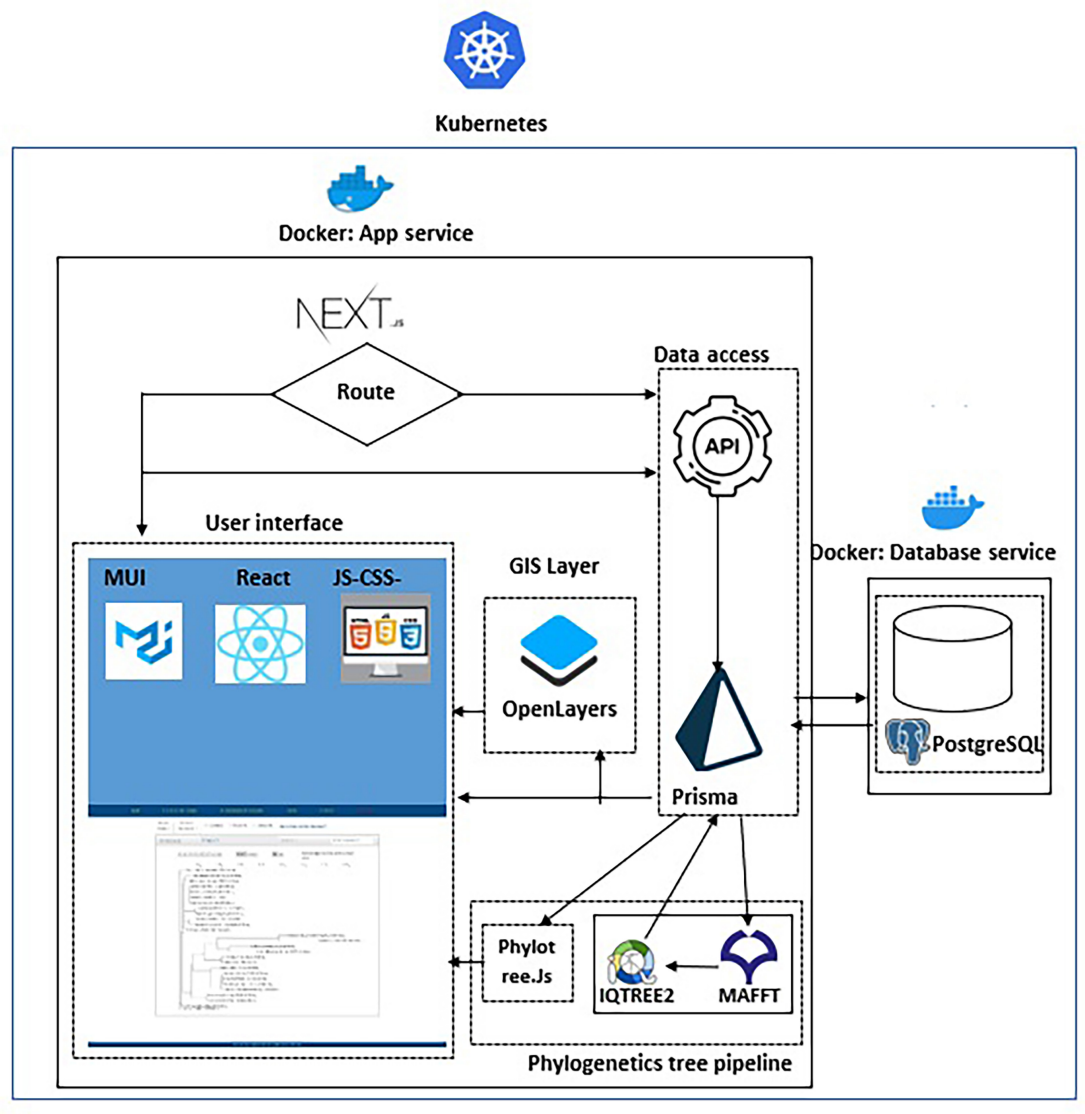
System Architecture: At the highest level of the architecture, Kubernetes is used to manage the system, denoted by the outer blueish line. Below Kubernetes, the app service built on Next.Js and the database service (data source) built on PostgreSQL are housed within Docker containers shown in continuous black border lines. The components within each service, are depicted as dotted lines. The API fetches data from the database to be displayed in the User interface. The GIS layer is used to display sampling regions on a map. MAFFT, IQTREE, and Phylotree.js have been integrated for phylogenetic tree construction and visualization.

For the system architecture, we adopted a microservices approach (defined in Table A1 in Appendix A) and containerization (defined in Table A1 in Appendix A) as shown in Figure 1. At the highest level of the system architecture, we leveraged Kubernetes, an open‐source container orchestration platform in deploying and managing the two microservices that constitute the application. Kubernetes enabled the deployment of the Docker containers (defined in Table A1 in Appendix A) for the platform in a shared computing environment that hosts other containers. Docker was used to build the containers for the application. Kubernetes was also adopted because it secures communication requests via the application programming interface (defined in Table A1 in Appendix A) by using an integrated software called NGINX (defined in Table A1 in Appendix A).

#### 2.2.1 Microservices

Within Kubernetes, the system architecture consists of two main microservices, the app service, and the database service. The app service functions as the front end (defined in Table A1 in Appendix A) layer of the system and is containerized using Docker, providing a lightweight and consistent runtime environment. The database service acts as data storage and is also containerized for efficient data storage and retrieval. The communication between the app service and the database service is facilitated by Kubernetes.

#### 2.2.2 Database component

The database design began with an analysis of the system requirements and entities involved, such as wild plants, fruits, and host insects. The next step was to design the database using standard methodology as described by Teorey et al. (1986). The PostgreSQL (PostgreSQL, 2023) database management system (DBMS) was adopted for data storage due to the structured nature of the biocollections dataset. Additionally, PostgreSQL supports a wide range of data types such as geographic location data which was part of the biocollection records. The database was implemented using structured query language (SQL). The entity‐relationship (ER) diagram (defined in Table A1 in Appendix A) for the database is available on GitHub (icipe, 2023f). The SQL structure and queries used for creating database tables are also available on GitHub (icipe, 2023e).

#### 2.2.3 Data preprocessing and migration

The data about plant species, fruiting months, insect codes, image codes, region codes, their morphological features, taxonomy, as well as insects reared from them, were contained in a master Excel file. Additional information, such as insect names mapped to the codes in the master file, collection regions, and glossary terms, was stored in separate Excel files. Images of plants, fruits, and insects were also in separate files. To perform data cleaning, Python scripts were developed (Appendix S1) and are available on GitHub (icipe, 2023d). The scripts were tailored to remove duplicates and address inconsistencies in the data. Subsequently, the data was structured into SQL tables, aligning with the tables outlined in the ER diagram. The SQL tables were loaded into the database using psql, which is the interactive terminal for PostgreSQL. The barcode data corresponding to the biocollections were downloaded analyzed separately and saved as a comma‐separated value (CSV) file. The importation process for barcodes into SQL tables was achieved using psql.

#### 2.2.4 Molecular data integration

For molecular data integration into the local database, we used the identified species names that matched the barcodes of the sequences from BOLD (Ratnasingham & Hebert, 2007). The BOLD‐API (BOLD, 2023) was used in tailored Python scripts stored in GitHub (icipe, 2023c). The database was accessed on 13 August 2023. Maturase K (matK) and cytochrome oxidase subunit I (COI) barcode sequences were downloaded and analyzed in custom Python scripts (Appendix S1). During the downloading process, various metadata fields were downloaded including the type of marker, country of origin, BOLD specimen ID, and NCBI accession number.

The preliminary quality control was performed by identifying the barcodes that were flagged to be of poor quality by BOLD. Further quality check involved analysis of Kimura 2‐parameter (k2P) genetic distance using 0.02 (Hebert et al., 2003) threshold and length filtering at a threshold of 400 base pairs (Kress & Erickson, 2008). To detect questionable barcodes, we performed the intraspecific distance evaluation using K2P metric while leveraging MEGA software (Kumar et al., 1994). The sequences with either intraspecific distance greater than 2% threshold or had only one barcode represented were, subjected to further evaluation through NCBI‐BLAST to identify questionable barcode sequences (Johnson et al., 2008; Meiklejohn et al., 2019; Pentinsaari et al., 2020). The steps for the phylogeny pipeline are illustrated in a documentation in GitHub (icipe, 2023h).

#### 2.2.5 Application programming interface (API) component

The API development relied on Prisma (defined in Table A1 in Appendix A) to connect with the underlying database to retrieve the necessary data. The documentation of the APIs is accessible on GitHub (icipe, 2023a), their types, and the purpose of each API.

#### 2.2.6 User interface

The user interface makes the data from the back end (defined in Table A1 in Appendix A) to be available to users for interaction at the front end. As shown in the system architecture, the data is retrieved from API, and if geographical information data, it is passed through the OpenLayers, which make up the geographical information system (GIS) component that renders the geographic coordinates on the map to users. On the other hand, the phylogenetic data is rendered via a phylogenetics tree component made up of MAFFT (Katoh & Standley, 2013) and IQTREE2 (Minh et al., 2020). The phylogenetic tree is rendered to the front end as Newick format (Olsen, 1990) and interactive visualization using Phylotree.js (Shank et al., 2018). The other types of data such as plant morphology, fruiting months, and insects reared from fruits data are being rendered directly to the front end using the reusable components of Next.js (Thakkar & Thakkar, 2020). The reusable components were implemented using the material user interface (MUI) (MUI, 2023).

### 2.3 Deployment

The deployment of the website was facilitated through the utilization of Kubernetes (Kubernetes, 2023), Docker (Merkel, 2014), and GitHub (Github, 2023). The website is hosted here – https://test‐dmmg.icipe.org/wpfhi. In the future users will be redirected to the new URL link.

## 3 RESULTS

### 3.1 Barcode retrieval

The results of the barcode retrieval and analysis are presented in Table 1. Among the 873 plant records, 267 species were found to have matK barcodes. Among these matK barcodes, 21 species showed intraspecific distances greater than the 0.02 K2P threshold. Additionally, two barcode sequences from plants were identified as low‐quality from the BOLD database, and 26 barcodes were less than 400 base pairs in length. Additionally, 73 plant species showed a lack of divergence in their barcodes. In the case of insects, for the 595 records, 183 of these had taxonomy records of identification to species level. The identified insect species were used for retrieval of relevant barcodes, resulting in 87 species with retrievable barcodes. Among these, 20 insect species exhibited intraspecific distance greater than 0.02 K2P threshold. Only 1 insect barcode was identified as low‐quality from BOLD, and 5 barcodes were less than 400 base pairs in length. The length of matK barcodes for plants was in the range of 205–913 base pairs. On the other hand, the insect's COI length was in the range of 235–888 base pairs indicating the need for filtering based on barcode length.

**TABLE 1:** Barcode retrieval and analysis for plants and insects.

Organism	*N*	Barcode type	*N* with barcodes	*N* with K2P > 0.02	Total barcodes	<400 bp	No divergence	Barcode length range
Plants	873	matK	267	21	685	26	73	205–913 bp
Insects	183	COI	87	19	715	5	2	235–888 bp

The table shows the number of barcodes retrieved, barcode types retrieved, species with barcodes, species with K2P greater than 2% genetic distance, total barcodes retrieved, barcodes less than 400 bp, species with no divergence, and barcode length range.

### 3.2 Functionalities of the WiPFIM digital platform

#### 3.2.1 Browsing plants and fruits with insects

**FIGURE 2:**
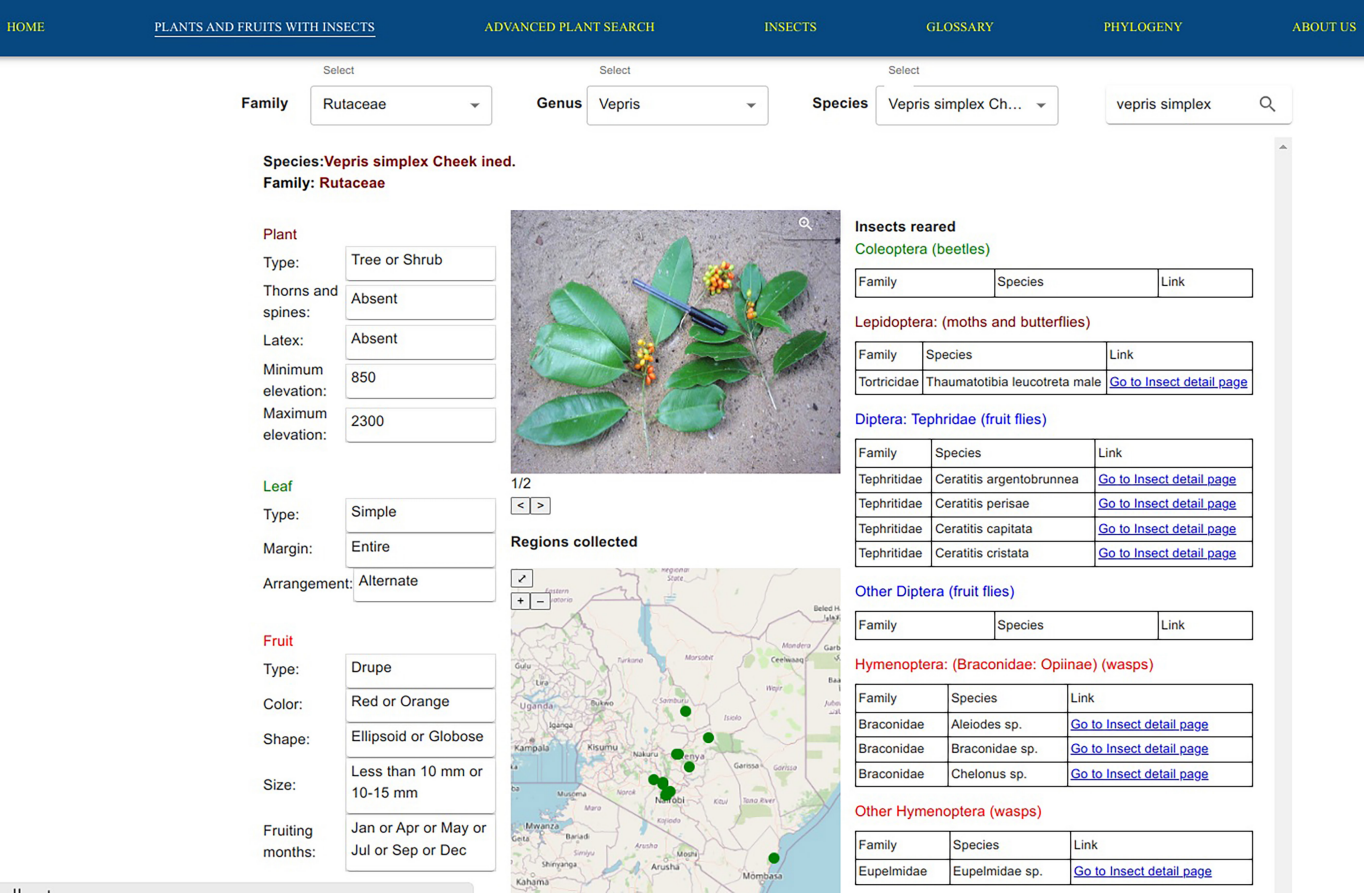
Plants and fruits with insects web page describing *Vepris simplex*. The plant has both orange and red fruits when ripe. Fruits of this plant were sampled in January, April, May, and December. The fruits of this plant have a wide range of associated insects, mostly Ceratitis species. One case of a Lepidoptera species was also found to feed on *Vepris simplex*. Three species of Braconidae, which are parasitoids of other insects, were reared from *V. simplex* fruits. These had probably attacked the moth species.

The results of the plants and fruits with insects web page can enable users to explore information about various plant species and fruits as shown in Figure 2. This system's web page provides information on the plants, insects reared from fruits, fruit shape, fruit size, fruit color, the regions of collection, images of fruits and plant specimens, fruiting months, leaf arrangements, leaf type, leaf shape, and other plant morphological descriptions. The feature also presents a functionality for users to search for information on plant species of interest. Additionally, the system presented an integration of insect data, providing a list of insect taxa reared from the selected plant species, with the ability of users to navigate to the details of each insect. The map on this web page shows regions across Kenya where the plant species were sampled. Some of the insects observed in the fruits may be parasitoids. However, it is not explicit which species are parasitoids and which are genuine fruit feeders in this platform, although many researchers may be able to deduce this information.

#### 3.2.2 Advanced plant search

From the advanced plant search web page, the system provides users with the possibility to identify an unknown plant species using morphological features such as plant type, presence or absence of latex, fruit color, fruit shapes, fruit sizes, fruit types, leaf types, leaf arrangements, and leaf margins. After selection, users can send the query to the database using the submit button to retrieve the results with a list of plant species that match the query and description of the plants. In this functionality, users can also clear any selected terms using the clear button. When the user selects a combination of plant features that are not present in the database, they will be notified. If a user doesn't know the meaning of a term double‐clicking on it will take the user to the glossary entry for that term. However, this feature is only possible for fruit type, fruit shape, and leaf features. In the glossary page, images of plants or plant parts that illustrate the term in question appear along with the meaning.

#### 3.2.3 Browsing insect data

**FIGURE 3:**
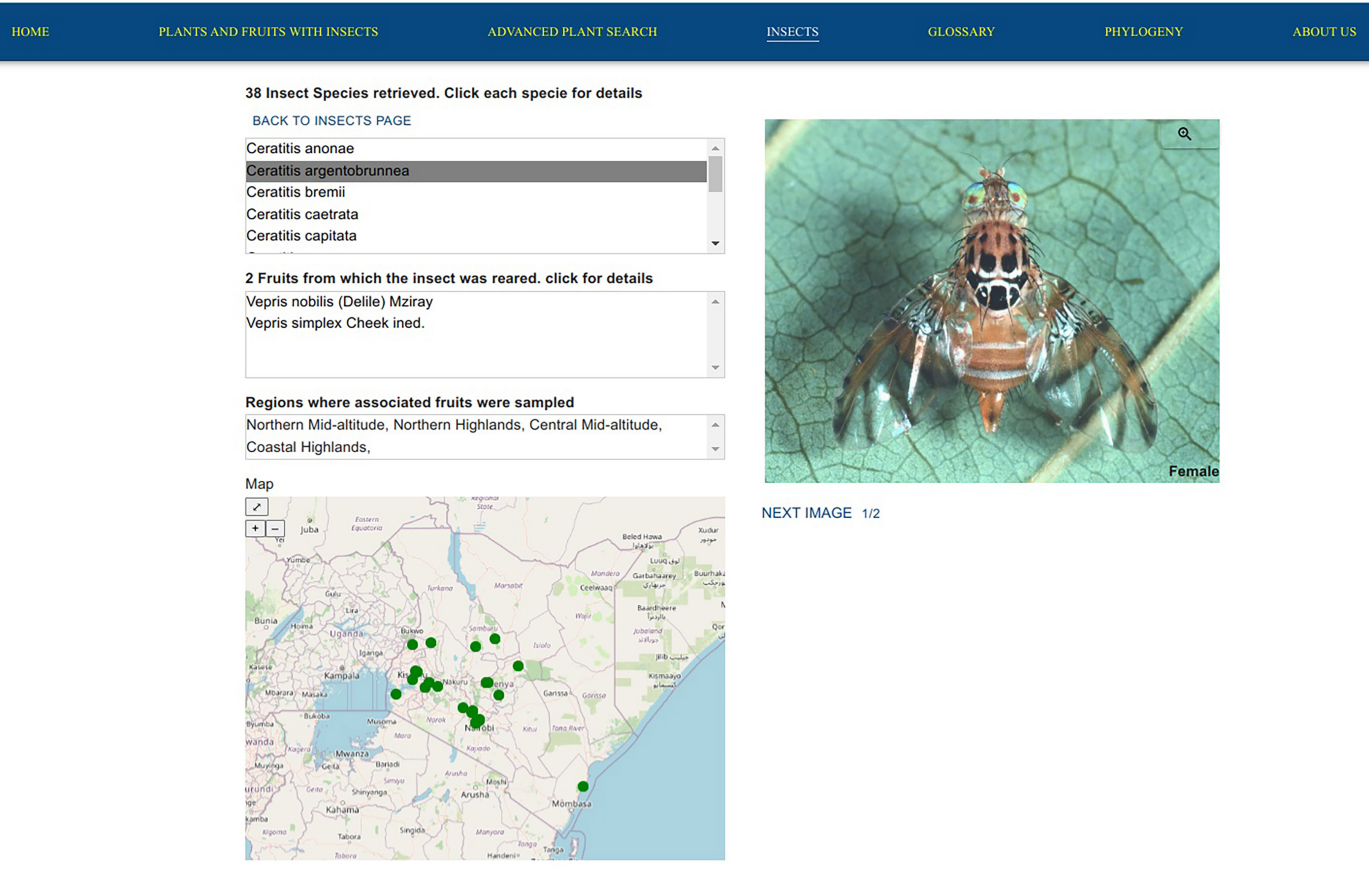
Insect web page describing the genus Ceratitis. The list of species under the genus is shown. The species are arranged alphabetically. In this figure, the first species shown is *Ceratitis argentobrunnea*. The information includes associated plants, image(s) sex, and sampling regions of the fruit that yielded *C. argentobrunnea*.

The results in Figure 3 show the information contained in the insect web page. Users can explore insect records including their images, associated fruits, sex (if it was determined), distribution, and other related insect species within the genus selected. Users can navigate to this web page after selecting the genus of interest from the insects' home web page which contains information on insect taxonomy from order level to genus level.

### 3.3 Interactive web phylogeny

**FIGURE 4:**
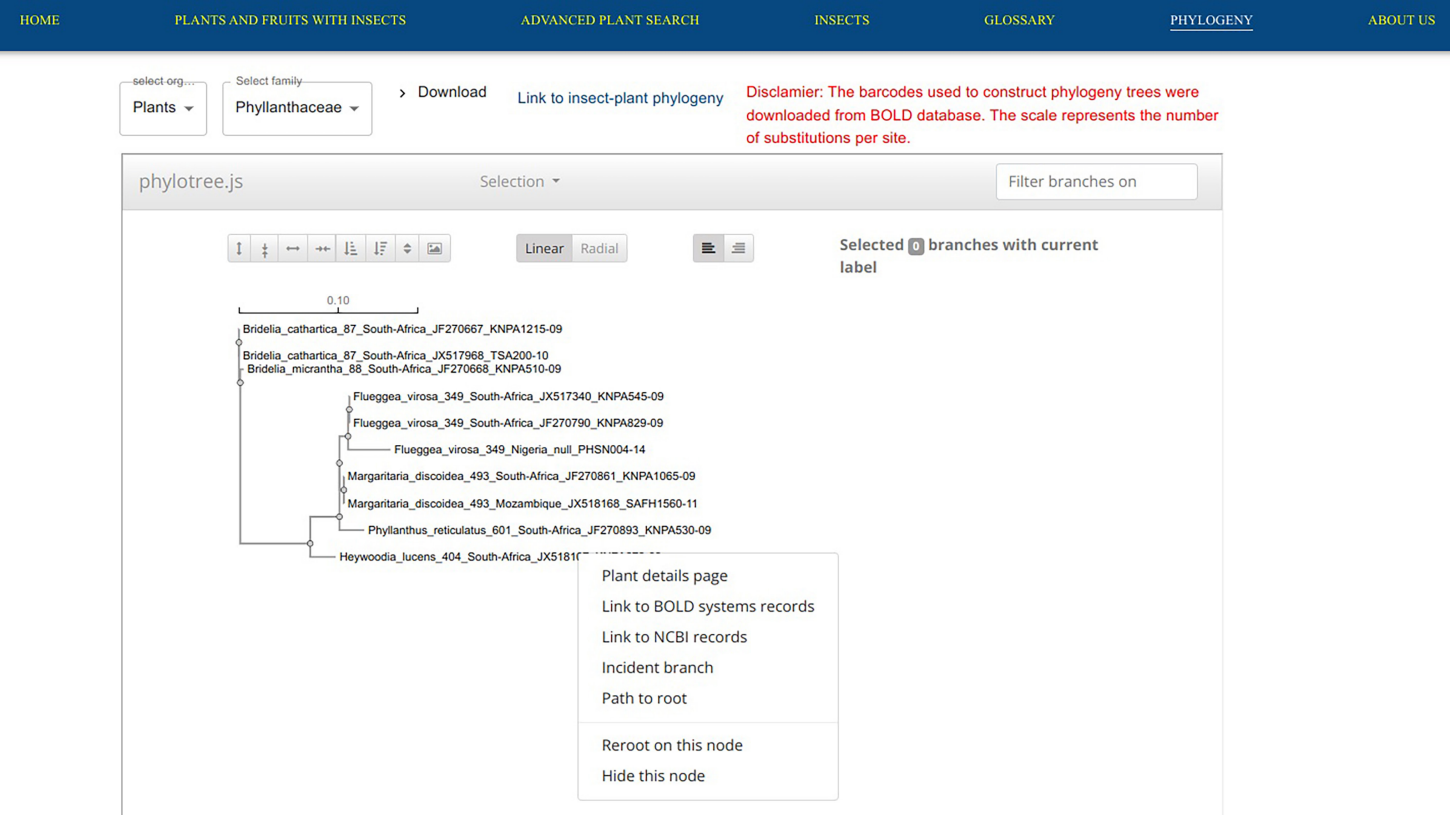
Phylogeny page for Phyllanthaceae plant family. The label of the terminal taxa consists of the species name, followed by the species ID as retrieved from the local database. The next part is NCBI accession and the last part is BOLD. The null at the last part of the name shows the absence of the NCBI accession number for the respective species from the BOLD database. Some species like *Margaritaria discoidea* species showed no genetic variation for the represented species. On the other hand, *Flueggea virosa* shows genetic divergence within the species. The scale represents substitutions per site per site. Left‐clicking each terminal taxa brings more options for users.

The results in Figure 4 show the phylogenetics functionalities of the digital platform. The phylogeny web page provides users with the ability to visualize the phylogenetic trees of plants and insects based on their barcodes grouped into families. However, only plant and insect families with barcode data are shown on the phylogeny page. In addition, the feature contains a link of only insects that had both barcode data and associated plants known included in the labeling (icipe, 2023g). As a disclaimer, we did not do barcoding for the work, therefore, we reused the existing barcodes from BOLD (Ratnasingham & Hebert, 2007) with the assumption that the identifications of the biocollections were correctly done.

Through phylotree.js, the platform enables users to extend the phylogenetic tree both vertically and horizontally, providing an interactive view of evolutionary relationships. Additionally, users can selectively color and highlight specific branches of the tree. Also, users can filter terminal taxa by typing species names in the search section which highlights the branches with the matching species. Through the Phylotree.js feature, users can collapse a subtree or trace path to the root by clicking on the node to see these options among others. The platform offers the possibility to download the Newick tree format or barcode sequences from which the phylogenetic tree was constructed. The option for displaying the tree as radial or linear is also possible from this page.

## 4 DISCUSSION

### 4.1 Database structure

Researchers can utilize this database to access information on plants, fruits, and associated insects. The database comprises mostly woody plants which usually produce larger numbers of fruits compared to herbaceous plants. Since certain fruit fly groups do not feed on fruits but on flowering parts, whereas others may eat flower and fruit parts, representatives of these plant families are also represented in this digital platform.

### 4.2 Molecular barcode retrieval

It was observed that some species exhibited intraspecific distances greater than 2%, indicating potential issues with species identification or the presence of cryptic species. However, some of the observed genetic divergence could be due to the diverse geographic distributions (Gaytan et al., 2020). The species‐level identification inconsistencies observed could be attributed to the inclusion of misidentified specimens in public databases (Meiklejohn et al., 2019). By applying quality control strategies including length filtering, intraspecific distance evaluation, and conducting BLAST analyses for validation, we ensured that accurate barcodes were integrated into the local database. During the analysis, the barcodes that were assigned to different genera after NCBI‐BLAST were considered questionable, which could mean potential errors in barcode identification.

### 4.3 Morphological and biogeographic data integration

The primary function of the system was to enable users to explore information about plants and fruits. In the plants and fruits with insects web page, some of the insects observed in the fruits may be parasitoids. However, it is not explicit which species are parasitoids and which are genuine fruit feeders in this platform, although many researchers may be able to deduce this information. The home web page has a search option, allowing users to search for specific plant or insect species details by typing the species name.

This platform contains plant records specifically collected by Copeland et al. (2009) during the fruiting months of plants which were determined empirically. This targeted approach acknowledges the significance of this period for species identification. Using fruit and plant morphology as field identification markers and optimizing collection timing. Plants are easier to identify when flowering or fruiting and, whereas flowering specimens are the cornerstone of plant taxonomy, fruits are often available when flowers are not. These two features of plants complement each other, greatly expanding the season when plants may be readily identified in the field (Hassoon et al., 2019).

Visual representations are essential in identifying plants in the field. The WiPFIM platform provides a collection of images for both fruit and plant specimens. This digital platform can aid in identifying plant species by selecting morphological characteristics (absence or presence of latex, woody or herbaceous, presence of thorns, spines and priddes, leaf type, leaf margin, leaf arrangement, and fruit type, size of the fruit) in the advanced plant search web page. Novice users can access the meaning of the terms on the glossary web page.

### 4.4 Molecular data linkages

Molecular data integration was based on the assumption that DNA barcodes are universally conserved (CBOL Plant Working Group1 et al., 2009) and that the individuals of the same morphospecies will have similar barcode sequences for matK and COI. Therefore, unsequenced individuals with a morphological identification to species level were assigned to the haplotype (DNA barcode sequence) corresponding to sequenced individuals with the same morphological identification in line with the study by Heckenhauer et al. (2017).

In the phylogeny page, we have included a link to insects and plants phylogeny, which represents all the insect barcodes that had an associated plant name, which is included in the labeling and can be useful to identify patterns of specialization and insect diversification in insect feeding on fruits (Jurado‐Rivera et al., 2008; Kergoat et al., 2017; Novotny et al., 2010). For example, the two insect species, *Trirhithrum meladiscum* and *Trirhithrum senex*, show an association with plants within the Rubiaceae plant family.

Several studies have used DNA barcodes to study plant–insect interactions (Bruzzese et al., 2019; Gougherty & Davies, 2021; Meiklejohn et al., 2019; Zhang et al., 2021). These studies have investigated insect‐feeding patterns as well as ecological niches. However, developing a digital framework to integrate phylogeny and fruit–insect interactions is complex and has been less explored. However, tools like SHOOT.bio (Emms & Kelly, 2022) have shown the possibility of integrating phylogenetics tools such as MAFFT, IQTREE, and ete3 (Huerta‐Cepas et al., 2016) in a digital framework for protein orthologs phylogenetic analysis.

Users can explore the functionalities of the platform by navigating to the about us page and selecting the ‘user guide option’ (icipe, 2023j). The provided data in the WiPFIM platform may be used to construct binary interaction matrices (Hawes & Peres, 2014) which, although useful, are limited in terms of their ecological interpretation.

## 5 CONCLUSION

The development of the WiPFIM WiPtFruIM platform represents an important step in studying fruit–insect interactions and understanding plant–insect relationships. The digital platform provides researchers, educators, and nature enthusiasts with open access to data on wild plants, fruits, and the insects associated with them. The WiPFIM platform opens new possibilities for scientific exploration, classroom education, and bridging the existing gap of limited digital data integration of heterogeneous data from the biocollections of wild, plants, fruits, and associated insects in Kenya, and extension, providing linkage to related molecular data. The digitization and accessibility of biocollections contribute to the preservation of essential bioresources and facilitate their utilization by the scientific community. With its potential to aid in plant species identification morphologically, the platform can contribute to taxonomic studies.

## 6 FUTURE RECOMMENDATIONS

In the future, this platform can be integrated with well‐established platforms such as GBIF and iNaturalist to broaden its usage and complement them. Moreover, expanding the use of additional barcode markers such as internal transcribed spacer (ITS) and ribulose‐1,5‐bisphosphate carboxylase (rbcl) and using barcodes from other databases will address the limited availability of barcodes for certain species.

## AUTHOR CONTRIBUTIONS

**Bonface Onyango:** Data curation (equal); formal analysis (equal); methodology (equal); software (equal); validation (equal); visualization (equal); writing – original draft (equal); writing – review and editing (equal). **Kennedy Senagi:** Conceptualization (equal); data curation (equal); formal analysis (equal); funding acquisition (equal); investigation (equal); methodology (equal); project administration (equal); resources (equal); software (equal); supervision (equal); validation (equal); visualization (equal); writing – review and editing (equal). **Robert Copeland:** Conceptualization (equal); data curation (equal); formal analysis (equal); funding acquisition (equal); investigation (equal); methodology (equal); project administration (equal); resources (equal); supervision (equal); validation (equal); visualization (equal); writing – review and editing (equal). **John Mbogholi:** Formal analysis (equal); funding acquisition (equal); investigation (equal); methodology (equal); software (equal); supervision (equal); validation (equal); visualization (equal); writing – review and editing (equal). **Mark Wamalwa:** Investigation (equal); methodology (equal); software (equal); supervision (equal); validation (equal); visualization (equal); writing – review and editing (equal). **Caleb Kibet:** Formal analysis (equal); investigation (equal); methodology (equal); software (equal); supervision (equal); validation (equal); visualization (equal); writing – review and editing (equal). **Henri E. Z. Tonnang:** Conceptualization (equal); data curation (equal); formal analysis (equal); funding acquisition (equal); investigation (equal); methodology (equal); project administration (equal); resources (equal); software (equal); validation (equal); visualization (equal); writing – review and editing (equal).

## FUNDING INFORMATION

The authors gratefully acknowledge the financial support for this research by the following organizations and agencies: the Fogarty International Center at the National Institutes of Health under Award Number U2RTW010677; the Swedish International Development Cooperation Agency (Sida); the Swiss Agency for Development and Cooperation (SDC); the Australian Centre for International Agricultural Research (ACIAR); the Norwegian Agency for Development Cooperation (Norad); the German Federal Ministry for Economic Cooperation and Development (BMZ); and the Government of the Republic of Kenya. The views expressed herein do not necessarily reflect the official opinion of the donors.

## CONFLICT OF INTEREST STATEMENT

The authors declare that there are no conflicts of interest.

## Supporting information


Appendix S1

## Data Availability

The data is publicly accessible (through search and navigating on the website) within the digital platform website (icipe, 2023i). The associated code is available on our GitHub (icipe, 2023k).

## APPENDIX A

**TABLE A1:** Glossary of terms.

Term	Description
API	A mechanism that allows two independent applications to talk to each other
Back end	The infrastructure and data that make the application work
Containerization	The process of packaging an application and its dependencies into an isolated container
Docker	A platform designed to help developers build, share, and run container applications
Entity	A real‐world object that is represented in the database for example plants, insects, fruits, and sampling regions
ER diagram	A diagram that represents relationships among entities in a database
Front end	Part of the application that users see and include visual elements like checkboxes, buttons, graphics, and text messages that allow your users to interact with the application
Microservices	A software development approach where an application is composed of small independent services that communicate with each other through well‐defined APIs
Nginx	A tool that provides security of the APIs by redirecting HTTP requests to HTTPS requests
ORM	A method that lets you query and manipulate a database using only the programming language
Prisma	An ORM tool with which to build a REST API, which we used for efficient data retrieval
