# Supplementary material for: WiPFIM: A digital platform for interlinking biocollections of wild plants, fruits, associated insects, and their molecular barcodes
Source: Ecol Evol. 2024 Jun 1;14(6):e11457. doi: 10.1002/ece3.11457 (PMC11143469; doi:10.1002/ece3.11457)
Supplement: Supplementary file 1 — Appendix S1 [file ECE3-14-e11457-s001.docx]

================================================================

Supplementary Figure 1: ER diagram:

================================================================

The Entity-Relationship (ER) diagram that represents the database structure and relationships between the biological entities can be accessible here - <https://github.com/icipe-official/Wild-Plants-Fruits-and-Host-Insects/blob/main/database/UML.png>

================================================================

================================================================

Supplementary Figure 2: Phylogeny pipeline:

================================================================

The phylogeny pipeline diagram illustrates the steps of barcode retrieval, analysis, and phylogenetic tree construction. The diagram can be accessed here - <https://github.com/icipe-official/Wild-Plants-Fruits-and-Host-Insects/blob/main/updated_phylogeny_workflow.jpg>

================================================================

================================================================

Supplementary Python scripts : Data preprocessing

================================================================

The scripts provide the steps for barcode retrieval, analysis, and phylogenetic tree construction. The Jupyter Notebook containing the scripts can be accessed here - <https://github.com/icipe-official/Wild-Plants-Fruits-and-Host-Insects/blob/main/notebooks/data_processing.ipynb>

================================================================

================================================================

Supplementary Python scripts: Barcode download

================================================================

The scripts contain the codes used for downloading barcode sequences from the BOLD. The Jupyter Notebook containing the scripts can be accessed here - <https://github.com/icipe-official/Wild-Plants-Fruits-and-Host-Insects/blob/main/notebooks/barcode_mining_from_BOLD.ipynb>

================================================================

================================================================

Supplementary Python scripts: Barcode analysis:

================================================================

These scripts contain analysis of the downloaded barcode sequences including quality control steps. The Jupyter Notebook containing the scripts can be accessed here - <https://github.com/icipe-official/Wild-Plants-Fruits-and-Host-Insects/blob/main/notebooks/barcode_ak2p_nalysis_and_qc.ipynb>

================================================================
